# Solid-state NMR [^13^C,^15^N] resonance assignments of the nucleotide-binding domain of a bacterial cyclic nucleotide-gated channel

**DOI:** 10.1007/s12104-012-9363-4

**Published:** 2012-02-03

**Authors:** Abhishek Cukkemane, Deepak Nand, Sabine Gradmann, Markus Weingarth, U. Benjamin Kaupp, Marc Baldus

**Affiliations:** 1 Bijvoet Center for Biomolecular Research, Utrecht University, Padualaan 8, 3584 CH Utrecht, The Netherlands; 2 Center of Advanced European Studies and Research (Caesar), Ludwig-Erhard-Allee 2, 53175 Bonn, Germany

**Keywords:** Cyclic nucleotide-binding domain, Cyclic AMP, Solid-state NMR, Magic-angle Spinning

## Abstract

Channels regulated by cyclic nucleotides are key signalling proteins in several biological pathways. The regulatory aspect is conferred by a C-terminal cyclic nucleotide-binding domain (CNBD). We report resonance assignments of the CNBD of a bacterial mlCNG channel obtained using 2D and 3D solid-state NMR under Magic-angle Spinning conditions. A secondary chemical shift analysis of the 141 residue protein suggests a three-dimensional fold seen in earlier X-ray and solution-state NMR work and points to spectroscopic polymorphism for a selected set of resonances.

**Electronic supplementary material:**

The online version of this article (doi:10.1007/s12104-012-9363-4) contains supplementary material, which is available to authorized users.

## Biological context

Cyclic nucleotides (cNMPs) are important secondary messenger molecules that mediate a multitude of processes by activating several different proteins in the signalling cascade. These proteins share a conserved regulatory protein unit referred to as cyclic nucleotide-binding domain (CNBD). Most of the CNBD harboring proteins are allosterically modulated. Upon binding cNMPs the regulatory CNBD renders the protein in an active conformation. One such family of proteins which is regulated by cNMPs is ion channels (Kaupp and Seifert 2002). On binding cNMPs, the channels are activated resulting in an increase in membrane conductance. Although a considerable amount of information is available on the function of these channel proteins, the molecular events that relay ligand binding to channel gating are not well understood. A major limitation in our understanding of the channel function arises from the fact that ligand binding and channel activation are two separate events that are reciprocally coupled, i.e. ligand binding affects channel gating, and conversely, channel gating affects ligand binding.

Until recently, it was believed that cNMP-gated channels are allosterically modulated and more than one ligand molecule is required to gate open the channel. However, the bacterial mlCNG channel displays distinctively different channel functioning property in being non-cooperative (Cukkemane et al. 2007; Nimigean et al. 2004). This provides a facile system in understanding the binding–gating relationship. In order to comprehend the molecular mechanisms of activation, a structural understanding of the channel and the CNBD is a prerequisite. The crystal structure (Clayton et al. 2004) and the solution NMR (Schünke et al. 2009) data of the CNBD from mlCNG are available. In line with other cAMP binding proteins (Berman et al. 2005), the cNMP binding site of the CNBD comprises three α-helices and eight β-strands. The β-strands form a cavity resembling a flattened β-barrel like structure which is popularly referred to as β-jelly roll-like structure. The cNMP is bound within this cavity through a network of polar and hydrophobic interactions with a short 3_10_ helix and loop between strands β6 and β7. This region is commonly referred to as the phosphate binding cassette and is the most conserved region of the protein.

Solid-state NMR (ssNMR) offers means to monitor structural or dynamical aspects of soluble proteins under a variety of preparatory conditions (see, e.g., Seidel et al. 2005) and can be used to study membrane proteins (MPs) in a functional membrane setting. Previously, we showed that spectroscopic studies in MPs encompassing several hundred amino acids are facilitated by reference experiments using smaller protein constructs (Etzkorn et al. 2007, 2010). Importantly, resonance assignments in the solid state may differ from values obtained in solution due to differential protein mobility or intermolecular interactions (Seidel et al. 2009). Therefore we investigated the CNBD of mlCNG channel using solid-phase preparations paving the way for structural studies on the full-length channel.

## Methods and experiments

The CNBD (residues 214–355) was expressed as a GST-fusion construct in pGEX-2T vector here (Clayton et al. 2004; Cukkemane et al. 2007) in *E.coli* BL-21 (DE3) pLysE using standard ^15^N/^13^C labeled media. Overnight expression (20°C) of the transformed *E.coli* cells was induced at OD_600_ ~ 0.6 using 0.6 mM IPTG. The harvested cells were lysed in PBS buffer by sonication. The supernatant containing the GST-CNBD was separated from the cell pellet by centrifugation. The pellet was discarded and the supernatant was loaded on to a glutathione affinity column (Glutathione Sepharose-4B). After washing with 10 ml (per 1 ml column) of washing buffer, the CNBD part of the fusion protein was cleaved off by thrombin. 40 units of thrombin were added to 1 ml of Glutathione Sepharose-4B column to release ~10 mg of CNBD protein. Incubation was done overnight at 27°C. The CNBD was eluted by a washing step whereas the GST part remained bound to the column.

The purified protein was dialysed against 10 mM Tris, pH 8.0, 250 μM cAMP and 0.01% Na-Azide at room temperature. The dialysate was flash-frozen and lyophilized in a 15 ml falcon tube (Greiner Bio-one). The lyophilized sample was rehydrated by placing the protein powder in a water bath for 3 days in an assembly that included a 250 ml conical flask with ~20 ml of water. The lyophilized sample was incubated in the flask, covered using parafilm at room temperature for 3 days during which the sample hydrated with a gel consistency. Around 10 mg of the rehydrated sample was packed in a volume of ~35 μl in a 3.2 mm zirconium rotor by centrifugation.

All ssNMR experiments were conducted using a 3.2 mm triple-resonance (^1^H,^13^C,^15^N) probe head at static magnetic field of 16.4T corresponding to 700 MHz proton resonance frequency (Bruker Biospin). Assignments were obtained using a combination of 2D and 3D correlation experiments. These experiments included 2D [^13^C,^13^C] correlations obtained using proton-driven spin diffusion under weak coupling conditions (Seidel et al. 2004) with spin diffusion times of 20 and 150 ms to encode intra-residue and sequential correlations, respectively. Furthermore, 2D [^13^C,^13^C] DQ-SQ and 3D [^13^C,^13^C,^13^C] DQ-SQ-SQ (Heise et al. 2005) were performed. SPC5 (Hohwy et al. 2002) was used for the generation of double quantum coherence with a contact time of 800 μs. 2D [^15^N,^13^Cα/C′] (Baldus et al. 1998) spectra were recorded using SPECIFIC-CP time of 3 ms. 2D [^15^N,^13^Cα–Cx] and 2D [^15^N,^13^C′–Cx] were recorded with ^13^C–^13^C mixing times of 40 and 50 ms, respectively. All the above mentioned experiments were performed at 10.127 kHz MAS at −10°C with SPINAL 64 (Fung et al. 2000) decoupling on ^1^H while acquiring the FID. The 3D [^15^N,^13^C′–Cx] experiment was performed at 17 kHz with 50 ms ^13^C–^13^C MIRROR recoupling (Scholz et al. 2008) and XiX (Detken et al. 2002) decoupling on ^1^H during acquisition. Chemical shifts were referenced to the upfield peaks of 31.48 and 38.9 ppm for ^13^C and ^15^N using Adamantane and tri-peptide AGG, respectively.

## Assignments and data deposition

**Fig. 1:**
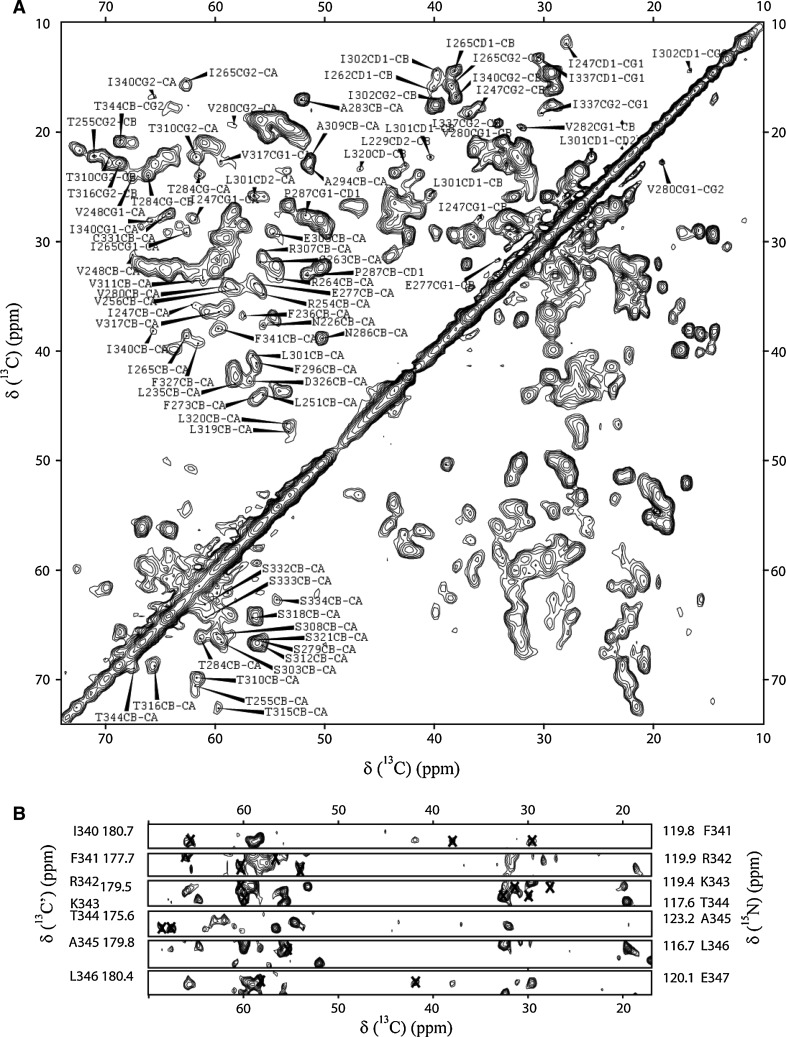
Characterization of the CNBD by ssNMR using (**A**) 2D [^13^C–^13^C] and (**B**) 3D [^15^N,^13^C′–Cx] correlation spectra. Intra-residue and sequential correlations are indicated in **a** and **b** respectively. Most signals assigned in the 2D [^13^C–^13^C] spectrum reflect amino acids that reside in the core region rich in β-strands (see Fig. 2A, B). The signals for residues in α-helices were resolved in a 3D [^15^N,^13^C′–Cx] spectrum. The strip plot shown in **B** contains correlations of residues located at the C-terminal helix of the protein

Backbone and side-chain chemical shifts of the CNBD are presented in Table S1 and have been deposited in the BioMagResBank (http://www.bmrb.wisc.edu) under the accession number 18024. Figure 1A, B display results of a 2D [^13^C–^13^C] spin diffusion and a 3D NCOCA correlation experiment obtained on uniformly [^13^C,^15^N] labeled CNBD, respectively. Intra-residue and sequential correlations are indicated. Overall, the line-width observed in both ^13^C and ^15^N dimensions compared favorably to results obtained previously in microcrystalline proteins (Seidel et al. 2005). Most signals assigned in the 2D spectrum reflect amino acids that reside in the core region rich in β strands (see Figure 1A/S1). Signals for residues in α-helices were resolved when using 3D ssNMR experiments. As an example, the strip plot shown in Fig. 1b contains correlations of residues located at the C-terminal helix of the protein. Resonances of five residues in the extreme N- and C-terminal parts of the CNBD were not identified. The Cα chemical shifts of residues of V224, Q228, L229, V23, A242 and E336 were not identified. Apart from those, the N chemical shifts of most of the Prolines and residues R225, Q237, V243, E267 and I337 were not assigned.

**Fig. 2:**
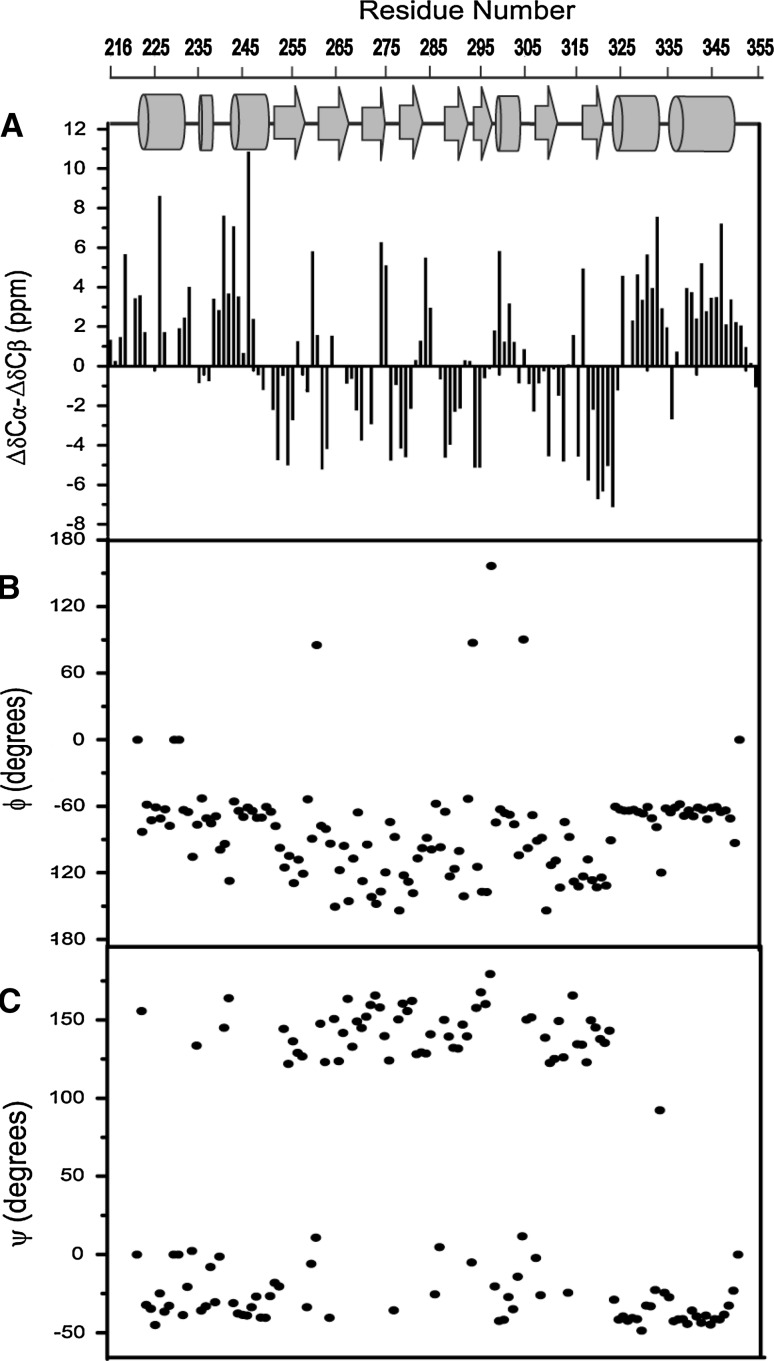
The pictorial representation of the secondary structure elements of the CNBD is shown in the *top* *panel* of **A**. Experimentally observed chemical shift parameter (δCα–δCβ) (Luca et al. 2001) in **A** of the CNBD compared against the derived backbone torsion angles using the program TALOS+ (Shen et al. 2009) in **B** and **C**

We obtained an assessment of the secondary structure and the overall fold of the CNBD using chemical shift differences (Fig. 2a). The backbone chemical shift of the CNBD is comparable to the results of the protein in solution (Figure S2 and S3D). Deviations between ssNMR and solution-state NMR assignments were found throughout the protein sequence (Figure S2) with largest differences generally detected towards the N and C terminus. Nevertheless, a secondary chemical shift analysis (Fig. 2B) revealed a backbone fold similar to that of previous crystal (Clayton et al. 2004) and solution-NMR (Schünke et al. 2009) studies of the cAMP-bound structure (Figures S3). Further analysis on the secondary structure was performed by predicting the dihedral angles ϕ and φ (Fig. 2B, C) with the program TALOS+ (Shen et al. 2009). Comparison of the TALOS+ predicted torsion angles between the ssNMR results and solution NMR (Figure S3A and S3B) show a profile indicating similar protein secondary structure. Overall, the chemical shifts of the CNBD determined by ssNMR results indicate a well folded protein. We obtained assignments for 86% of backbone residues with missing assignments predominantly found at both protein termini (Cα shifts of V224, Q228, L229, V233, A242 and E336). These parameters are, together with amino-acid side chain assignments, given in table S1.

Interestingly, we observed peak doublings in several regions of the protein (shown in Figure S3D), including loop residues connecting β1–β2, β2–β3, β7–β8 as well as the segment β6-PBC, and the N-terminal end of αC. Such spectroscopic polymorphism suggests the presence of multiple conformations in our ssNMR preparations even in the absence of the lipid-embedded transmembrane domain. Hence, our results on the 141 amino-acid CNBD will provide a valuable reference for correlating structural and dynamical aspects of the full-length channel to ligand binding in mlCNG in a membrane setting, and also aid in developing ssNMR methodology.

## Electronic supplementary material

Below is the link to the electronic supplementary material.

Supplementary material 1 (DOC 728 kb)
