# Supplementary material for: Solid-state NMR [13C,15N] resonance assignments of the nucleotide-binding domain of a bacterial cyclic nucleotide-gated channel
Source: Biomol NMR Assign. 2012 Feb 3;6(2):225–9. doi: 10.1007/s12104-012-9363-4 (PMC3438399; doi:10.1007/s12104-012-9363-4)
Supplement: Supplementary file 1 — Supplementary material 1 (DOC 728 kb) [file 12104_2012_9363_MOESM1_ESM.doc]

**Supporting Information:**

**
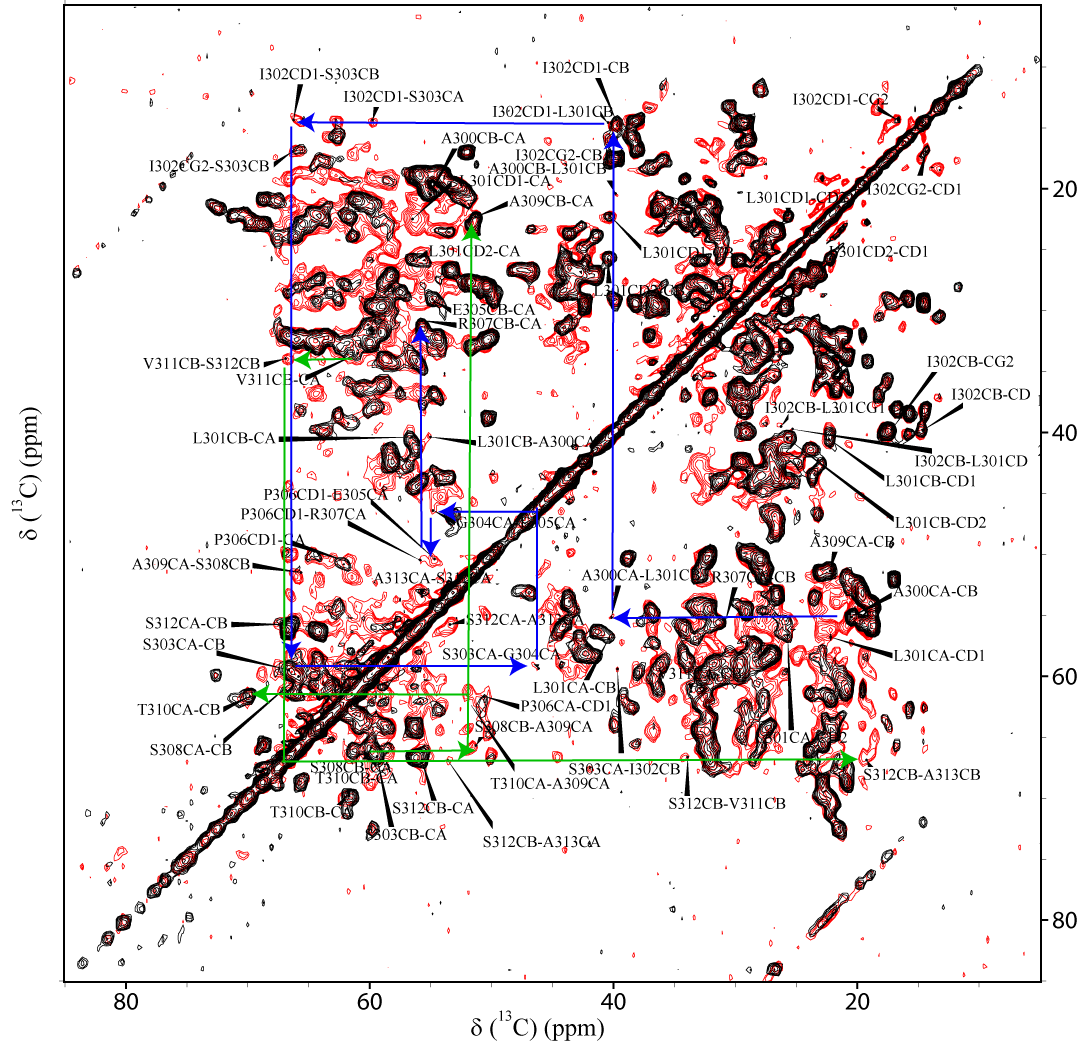
**

**Figure S1**: Comparison of 2D ^13^C-^13^C correlation spectra of the CNBD for short (20 ms, black) and longer (150 ms, red) mixing times. Connecting lines highlight a sequential walk for amino acid residues located in the Phosphate Binding Cassette (blue: A300-R307 and green S308-A313) of the protein.


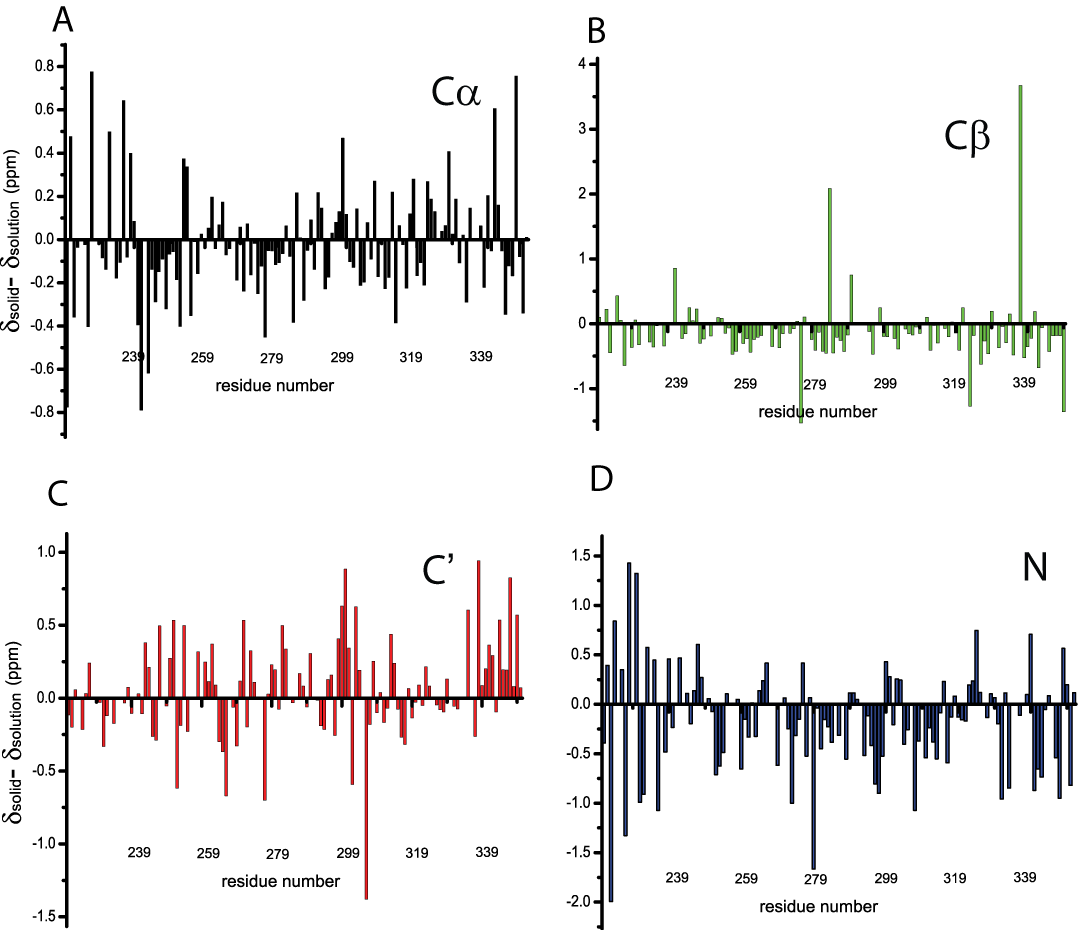


**Figure S2**: Comparison of the difference in (A) Cα, (B) Cβ, (C) C’ and (D) ^15^N-backbone chemical shifts (CS) obtained on soluble and solid-phase CNBD.


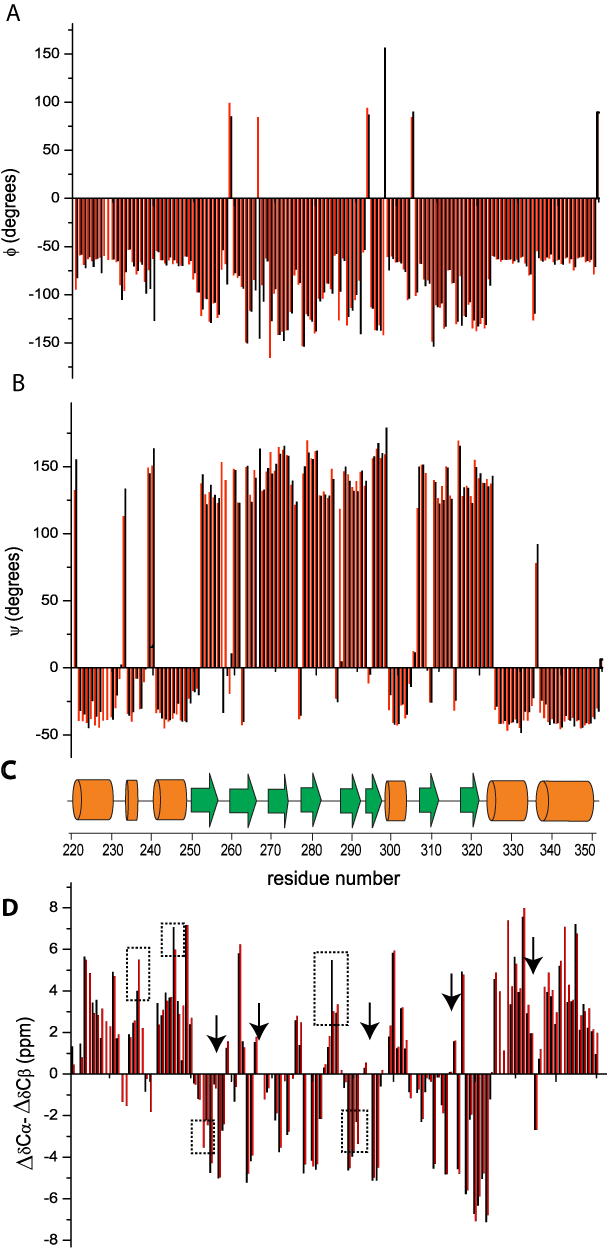


**Figure S3**: Analysis and comparison of backbone dihedral angles extracted for the CNBD from solution and solid-state NMR data. The solution NMR data (Schunke et al., 2007) are represented in red, while the solid-state NMR data are given in black. (A) and (B) are the predicted torsion angles for the CNBD using the TALOS+ (Shen et al., 2009) program. (C) summarizes the secondary structure elements of the CNBD. (D) depicts secondary chemical-shift parameters (Luca et al., 2001), which were calculated from the chemical shift assignments of the CNBD against the random coil value in solution NMR (Wang and Jardetzky, 2002). Protein regions exhibiting ssNMR peak doubling and chemical-shift changes compared to the solution-state data have been indicated by arrow and dotted boxes, respectively.

**Table S1**: Solid-state NMR Chemical shifts of CNBD. Numbering starts at the C-terminal end that follows the last transmembrane domain of the full-length mlCNG. Regions of the protein where polymorphism or large CS changes were observed have been indicated as * and **, respectively.

|  | CA | CB | CD1 | CD2 | CE1 | CG1 | CG2 | CO | N |
| --- | --- | --- | --- | --- | --- | --- | --- | --- | --- |
| R220 | 57.6 | 30.84 | - | - | - | - | - | - | - |
| G221 | 45.3 | - | - | - | - | - | - | 174.3 | 108.8 |
| D222 | 55.39 | 40.67 | - | - | - | - | - | 180.2 | 121.2 |
| F223 | 60.13 | 39.75 | - | - | - | - | - | 176.3 | 118.8 |
| V224 | - | - | - | - | - | - | - | - | 119.5 |
| R225 | 58.27 | 29.46 | - | - | - | - | - | 177.9 | - |
| N226 | 55.71 | 37.7 | - | - | - | 177.9 | - | 175.7 | 119.1 |
| W227 | 59.54 | 29.46 | - | - | - | - | - | 178.1 | 118.9 |
| Q228 | - | - | - | - | - | - | - | - | 116.2 |
| L229 | - | 42.62 | - | 22.78 | - | - | - | - | - |
| V230 | 65.25 | 31.28 | - | - | - | - | 22.47 | 177.9 | 116.1 |
| A231 | 54 | 18.64 | - | - | - | - | - | 177.6 | 119.7 |
| A232 | 51.76 | - | - | - | - | - | - | 176.5 | 117.2 |
| V233 | - | - | - | - | - | - | - | - | 120.3 |
| P234 | 65.91 | 32.59 | 52.95 | - | - | 27.01 | - | 177.7 | - |
| L235 | 58.27 | 42.97 | - | - | - | - | - | - | 119.4 |
| F236** | 57.32 | 37.19 | - | - | - | - | - | - | 110.4 |
| Q237 | 57.82 | - | - | - | - | 34.06 | - | 176.9 | - |
| K238 | 55.9 | 33.05 | - | - | - | 25.41 | - | 176.8 | 114.7 |
| L239 | 54.3 | - | - | 26.52 | - | - | - | 176.9 | 119.4 |
| G240 | 44.69 | - | - | - | - | - | - | - | 108.9 |
| P241 | 66.81 | 31.13 | 49.97 | - | - | - | - | 178.4 | 134.8 |
| A242 | 54.74 | 18.27 | - | - | - | - | - | 180.1 | 118.8 |
| V243 | 65.34 | 32.3 | - | - | - | - | - | 178 | - |
| L244 | 58.51 | 41.85 | - | - | - | 27.19 | - | 178.2 | 120.9 |
| V245** | 67.14 | 31.49 | - | - | - | - | - | 177 | 114.6 |
| E246 | 58.73 | 29.28 | 184.1 | - | - | 35.75 | - | 173.4 | 117.1 |
| I247 | 61.92 | 35.92 | 11.72 | - | - | 27.84 | 17.73 | 178 | 120.5 |
| V248 | 67.48 | 31.17 | - | - | - | 24.05 | 21.82 | - | 120.9 |
| R249 | 58.64 | 30.67 | 43.77 | - | - | - | - | 176.7 | 115.6 |
| A250 | 52.96 | 19.77 | - | - | - | - | - | 178.9 | 120.3 |
| L251 | 55.7 | 43.95 | - | 27.88 | - | - | - | 177.6 | 119.8 |
| R252** | 54.3 | - | - | - | - | - | - | 175.1 | 118.9 |
| A253 | 51.37 | 20.47 | - | - | - | - | - | 177.4 | 128 |
| R254 | 55.55 | 34.65 | 43.63 | - | - | 26.55 | - | 174.6 | 120.7 |
| T255* | 61.87 | 70.51 | - | - | - | - | 22.16 | 174 | 121.6 |
|  |  | 70.12 |  |  |  |  |  |  |  |
| V256* | 58.26 | 34.16 | - | - | - | - | 20.7 | - | 127.5 |
|  |  |  |  |  |  |  |  |  | 129.3 |
| P257 | 62.12 | 33.18 | - | - | - | 27.42 | - | 175 | - |
| A258 | 53.64 | 18.87 | - | - | - | - | - | 178.9 | 120.4 |
| G259 | 44.87 | - | - | - | - | - | - | 174.3 | 110.8 |
| A260 | 52.36 | 19.69 | - | - | - | - | - | 177.3 | 123.3 |
| V261 | 63.69 | 32.45 | - | - | - | 22.73 | - | 176.3 | 121.6 |
| I262 | 63.38 | 40.11 | 15.95 | - | - | 29.06 | 17.87 | 176.5 | 129.1 |
| C263 | 55.88 | 31.94 | - | - | - | - | - | 171.1 | 112.3 |
| R264 | 54.49 | 33.36 | - | - | - | - | - | 174.1 | 124 |
| I265 | 62.5 | 38.44 | 14.2 | - | - | 29.02 | 15.58 | 174.3 | 123.9 |
| G266 | 45.36 | - | - | - | - | - | - | 173.7 | 112.2 |
| E267 | - | - | - | - | - | 36.62 | - | - | - |
| P268* | 63.04 | 32.22 | 50.56 | - | - | - | - | 177.5 | - |
|  | 62.6 |  |  |  |  |  |  |  |  |
| G269* | 44.81 | - | - | - | - | - | - | 171.8 | 108.8 |
|  |  |  |  |  |  |  |  |  | 110.3 |
| D270 | 53.05 | 41.73 | - | - | - | 180.7 | - | 174.8 | 121.8 |
| R271 | 53.99 | 32.33 | - | - | - | 23.74 | - | 173.5 | 113.4 |
| M272 | 54.05 | - | - | 17.73 | - | - | - | 172.4 | 117.5 |
| F273 | 56.53 | 44.12 | - | - | - | - | - | 173.8 | 113.2 |
| F274 | 56.61 | 41.35 | - | - | - | - | - | 175.7 | 117 |
| V275 | 63.75 | 31.67 | - | - | - | 21.98 | - | 175.7 | 123.5 |
| V276 | 64.57 | 34.04 | - | - | - | - | - | - | 128 |
| E277 | 56.22 | 34.04 | 175.7 | - | - | 31.71 | - | 173.5 | 116.5 |
| G278 | 44.32 | - | - | - | - | - | - | 170.4 | 112.5 |
| S279 | 56.48 | 66.57 | - | - | - | - | - | 174.6 | 105 |
| V280 | 58.65 | 34.17 | - | - | - | 21 | 19.24 | 173.8 | 113 |
| S281 | 56.36 | 64.12 | - | - | - | - | - | 173.5 | 114.9 |
| V282 | 61.18 | 32.08 | - | - | - | 19.54 | 19.1 | 176.5 | 128.8 |
| A283 | 52.08 | 16.99 | - | - | - | - | - | 179.7 | 131.3 |
| T284** | 61.44 | 64.12 | - | - | - | - | 23.88 | - | 114.4 |
| P285 | 66.37 | 32.03 | 53.66 | - | - | 27.16 | - | 177.7 | - |
| N286 | 50.28 | 38.89 | - | - | - | - | - | - | 113 |
| P287 | 64.34 | 33.05 | 51.71 | - | - | 27.6 | - | 176.2 | 138.2 |
| V288 | 60.67 | 36.45 | - | - | - | 22.36 | 22.32 | 173.9 | 119.6 |
| E289 | 54.45 | 32.09 | - | - | - | 37.05 | - | 175.8 | 126.1 |
| L290 | 53.51 | 43 | 26.21 | 26.5 | - | - | - | 175.6 | 124.7 |
| G291 | 43.2 | - | - | - | - | - | - | - | 110.6 |
| P292 | 63.93 | 31.88 | 50.28 | - | - | 50 | - | 178.1 | 137.8 |
| G293 | 45.81 | - | - | - | - | - | - | 173.4 | 113.9 |
| A294 | 51.48 | 23 | - | - | - | - | - | 175.9 | 121.9 |
| F295* | 54.16 | 43.88 | - | - | - | - | - | 174.2 | 112.3 |
|  |  | 44.67 |  |  |  |  |  |  |  |
| F296 | 56.27 | 41.32 | - | - | - | - | - | 174.5 | 111.2 |
| G297 | 44.8 | - | - | - | - | - | - | 172.2 | 107.4 |
| E298 | 56.8 | 28.33 | - | - | - | - | - | 177.7 | 118.9 |
| M299 | 60.39 | 32.57 | - | 16.21 | - | - | - | 179.4 | 119.1 |
| A300 | 55.17 | 20.32 | - | - | - | - | - | 181 | 123 |
| L301 | 56.65 | 40.33 | 22.78 | 25.68 | - | 26.71 | - | 178.9 | 114.9 |
| I302 | 63.38 | 39.73 | 14.41 | - | - | - | 17.14 | 176.8 | 118.3 |
| S303 | 59.5 | 66.36 | - | - | - | - | - | 176.6 | 112.2 |
| G304 | 46.21 | - | - | - | - | - | - | 174.1 | 112.4 |
| E305 | 54.72 | 29.25 | - | - | - | 36.75 | - | - | 120.4 |
| P306 | 61.87 | 32.42 | 50.57 | - | - | - | - | 177.6 | 135.1 |
| R307 | 55.73 | 30.94 | - | - | - | 28.04 | - | 178.8 | 115.5 |
| S308 | 60 | 65.78 | - | - | - | - | - | 173.9 | 118.3 |
| A309 | 51.38 | 22.2 | - | - | - | - | - | 174.4 | 120.3 |
| T310 | 61.7 | 69.78 | - | - | - | - | 22.32 | 173.7 | 118.2 |
| V311 | 61.09 | 33.57 | - | - | - | 21.02 | 20.9 | 174.7 | 127.3 |
| S312 | 55.77 | 66.74 | - | - | - | - | - | 172.4 | 121.4 |
| A313* | 53. 2 | 19.32 | - | - | - | - | - | 178 | 128.4 |
|  | 53.9 |  |  |  |  |  |  |  |  |
| A314 | 53.96 | 19.07 | - | - | - | - | - | 177.9 | 132.3 |
| T315* | 59.7 | 72.63 | - | - | - | - | 21.32 | 173.6 | 108.9 |
|  |  |  |  |  |  |  |  |  | 110.9 |
| T316 | 65.58 | 68.81 | - | - | - | - | 22.58 | 176.4 | 115.5 |
| V317* | 59.47 | 36.6 | - | - | - | 23.12 | 22.37 | 173.6 | 130.3 |
|  |  | 35.7 |  |  |  |  |  |  |  |
| S318 | 56.56 | 64.3 | - | - | - | - | - | 173.4 | 121.3 |
| L319* | 53.24 | 47.31 | 26.45 | - | - | - | - | 176.9 | 125.3 |
|  |  | 48.0 |  |  |  |  |  |  |  |
| L320 | 53.47 | 46.67 | 24.31 | - | - | - | - | 176.2 | 120.2 |
| S321 | 56.5 | 66.35 | - | - | - | - | - | 172.9 | 117.7 |
| L322 | 52.53 | 46.62 | 24.79 | 24.85 | - | 27.16 | - | 175 | 124.6 |
| H323 | 57.7 | 32.91 | - | - | - | - | - | 176.5 | 127.2 |
| S324 | 62.08 | 63.04 | - | - | - | - | - | 175.9 | 119.2 |
| A325 | - | - | - | - | - | - | - | - | 124.8 |
| D326 | 56.71 | 42.92 | - | - | - | - | - | 177.7 | - |
| F327 | 61.62 | 39.25 | - | - | - | - | - | 176.9 | 122.9 |
| Q328 | 58.6 | 27.98 | - | - | - | - | - | 178.7 | 119.8 |
| M329 | 58.97 | 31.3 | - | 17.55 | - | - | - | 178.9 | 120.4 |
| L330 | 58.23 | 41.3 | - | - | - | - | - | - | 123.2 |
| C331 | 64.04 | 27.26 | - | - | - | - | - | 176.3 | 113.7 |
| S332 | 60.86 | 63.12 | - | - | - | - | - | 175.7 | 113.7 |
| S333 | 60.85 | 64.42 | - | - | - | - | - | - | 114.6 |
| S334 | 54.45 | 62.66 | - | - | - | - | - | - | - |
| P335 | 64.78 | 32.39 | - | - | - | - | - | 179.6 | - |
| E336 | - | - | - | - | - | 36.72 | - | - | 117.3 |
| I337* | 63.67 | 33.04 | 17.23 | - | - | 30.46 | 18.33 | 177.3 | - |
|  | 64.46 |  |  |  |  |  |  |  |  |
| A338 | 56.19 | 18.81 | - | - | - | - | - | 181 | 122.6 |
| E339 | 58.47 | 29.69 | 180.1 | - | - | 35.45 | - | 178.4 | 116.8 |
| I340 | 65.62 | 37.96 | 14.38 | - | - | 28.03 | 18.94 | 180.6 | 120.4 |
| F341 | 59.9 | 37.94 | - | - | - | - | - | 177.7 | 119.8 |
| R342 | 60.23 | 31.34 | - | - | - | 27.62 | - | 179.5 | 118 |
| K343 | 59.84 | 32.45 | 29.86 | 42 | - | 25.9 | - | 179.4 | 119.4 |
| T344 | 67.67 | 68.73 | - | - | - | - | 20.81 | 176 | 117.4 |
| A345 | 55.64 | 19.48 | - | - | - | - | - | 179.8 | 123.7 |
| L346 | 58.15 | 42.17 | 24.97 | - | - | - | - | 180.5 | 116.7 |
| E347 | 58.3 | 29.71 | - | - | - | 35.93 | - | 180.5 | 120 |
| R348 | 58.97 | 31.49 | - | - | - | - | - | 177.4 | 120 |
| R349 | 58.39 | 31.99 | - | - | - | - | - | 178.6 | 119.7 |
| G350 | 45.68 | - | - | - | - | - | - | 174.4 | 106.1 |
| A351 | - | - | - | - | - | - | - | - | 123.6 |

**References:**

Luca, S., Filippov, D.V., van Boom, J.H., Oschkinat, H., de Groot, H.J., and Baldus, M. (2001). Secondary chemical shifts in immobilized peptides and proteins: a qualitative basis for structure refinement under magic angle spinning. Journal of biomolecular NMR *20*, 325-331.

Schunke, S., Novak, K., Stoldt, M., Kaupp, U.B., and Willbold, D. (2007). Resonance assignment of the cyclic nucleotide binding domain from a cyclic nucleotide-gated K(+) channel in complex with cAMP. Biomol NMR Assign *1*, 179-181.

Shen, Y., Delaglio, F., Cornilescu, G., and Bax, A. (2009). TALOS+: a hybrid method for predicting protein backbone torsion angles from NMR chemical shifts. Journal of biomolecular NMR *44*, 213-223.

Wang, Y., and Jardetzky, O. (2002). Probability-based protein secondary structure identification using combined NMR chemical-shift data. Protein Sci *11*, 852-861.
